# Effect of vitamin E with and without saffron on the sexual function in women of reproductive age with sexual dysfunction: a randomized controlled trial

**DOI:** 10.1186/s12905-024-02980-w

**Published:** 2024-02-26

**Authors:** Saeideh Izadi, Sakineh Mohammad-Alizadeh-Charandabi, Parvin Yadollahi, Mojgan Mirghafourvand

**Affiliations:** 1 Student Research Committee, Midwifery Department, Tabriz University of Medical Sciences, Tabriz, Iran; 2 Department of Midwifery, Faculty of Nursing and Midwifery, Tabriz University of Medical Sciences, Tabriz, Iran; 3 Maternal-fetal medicine Research Center, Department of Midwifery, School of Nursing and Midwifery, Shiraz University of Medical Sciences, Shiraz, Iran; 4 Social Determinants of Health Research Center, Tabriz University of Medical Sciences, Tabriz, Iran

**Keywords:** Saffron, Vitamin E, Sexual dysfunction, Women of reproductive age

## Abstract

**Background:**

Sexual satisfaction is a crucial part of a fulfilled life, and the ability to have satisfying sexual function is crucial to one’s sexual health. This study investigated the effect of the combined administration of saffron and vitamin E and vitamin E alone on the sexual function of women in their reproductive years.

**Methods:**

A triple-blind randomized controlled trial was conducted with 50 participants experiencing sexual dysfunction without comorbid sleep disorders or severe depression. They were allocated into two groups using a block randomization method (stratified based on the severity of moderate or mild/normal depression). During the 8-week intervention period, participants in the experimental group were administered a 15 mg saffron capsule (safrotin) in the morning and a combination capsule containing 15 mg saffron and 50 mg vitamin E (safradide) in the evening. During the same period, the control group consumed one saffron placebo capsule in the morning and one capsule containing 50 mg of vitamin E and saffron placebo in the evening (in identical appearance to safradide). The Female Sexual Function Index was used to assess sexual function, and the Depression, Anxiety, and Stress Scale-21 (DASS-21) was used to measure levels of depression, anxiety, and stress. These measures were administered at baseline as well as four and eight weeks post-intervention, with an additional measurement taken four weeks after the intervention ceased. The repeated measures ANOVA, ANCOVA, and Mann-Whitney U tests were used to compare the groups.

**Results:**

Following the intervention, the experimental group (saffron and vitamin E) demonstrated a statistically significant increase in the overall mean score of sexual function compared to the control group (placebo of saffron and vitamin E) (adjusted mean difference (AMD): 4.6; 95%CI: 3.1 to 6.1; *p* < 0.001). The mean scores for sexual function dimensions, namely libido, arousal, orgasm, and satisfaction, except for pain, were consistently higher than those of the control group across all time points (*p* < 0.001). Additionally, the mean score for lubrication was significantly higher only at the eighth-week measurement (*p* = 0.004). The mean depression score in the experimental group was significantly lower than in the control group at all-time points, i.e., four (*p* = 0.011) and eight weeks after the intervention (*p* = 0.005), and four weeks after the end of the intervention (*p* = 0.007). The experimental group exhibited a statistically significant decrease in mean anxiety score compared to the control group at four weeks into the intervention (*p* = 0.016) and four weeks following the end of the intervention (*p* = 0.002). At eight weeks post-intervention, however, there was no significant difference between the groups (*p* = 0.177). Additionally, the experimental group exhibited a significant reduction in the overall mean stress score compared to the control group after the intervention (AMD: -2.3; 95%CI: -3.1 to -1.5; *p* < 0.001).

**Conclusion:**

Using the combination of saffron and vitamin E is more effective in improving sexual function and its domains compared to vitamin E alone in women of reproductive age with sexual dysfunction without severe depression. Also, it diminishes the degree of depression, anxiety, and stress more compared to vitamin E alone. However, further research is required to arrive at a more definitive conclusion.

**Trial registration:**

Iranian Registry of Clinical Trials (IRCT): IRCT20100414003706N36. Date of registration: 17/05/2020; URL: https://en.irct.ir/trial/45992; Date of first registration: 21/05/2020.

## Background

Sexual satisfaction is a crucial part of a fulfilled life [1], and the ability to have satisfying sexual function is crucial to one’s sexual health [2]. A significant contributor to emotional tensions and marital problems is sexual dysfunction, which can negatively impact a couple’s quality of life, self-esteem, mood, and relationships, as well as cause anxiety and depression [3–5]. Forty to fifty% of women, regardless of age, report at least one sexual dysfunction [6].

One approach to treating sexual dysfunction is the use of herbal medications [7]. Crocus sativus, commonly referred to as saffron, is a small perennial plant belonging to the Iridaceae family cultivated in a few countries, particularly Iran. Crostin, crocin, and safranal are thought to be responsible for saffron’s therapeutic effects [8]. A recent meta-research review of meta-analyses has demonstrated the safety of this plant for medicinal use as well as its efficacy in enhancing various clinical outcomes [9]. A 2019 systematic review found only five trials involving a total of 173 male and female participants. It also noticed the positive effect of saffron on sexual function, although with high heterogeneity in the results [10]. Limited research has been carried out to demonstrate the favorable effect of saffron on sexual dysfunction among women of reproductive age [11], menopausal women [12], and women of reproductive age experiencing severe depression while undergoing fluoxetine treatment [13]. Additionally, saffron has been found to have no significant effect on the sexual desire of men and women aged 40 to 60 hospitalized with coronary heart disease [14].

Considering the effect of saffron on the symptoms of depression [15], blood pressure [16], and cognitive function [17], the results of studies on women with depressive disorder and postmenopausal women may not be generalizable to women of reproductive age without obvious symptoms of depressive disorder. In the only trial on sexual dysfunction in women of reproductive age, the state of depression was not investigated [11].

Vitamin E is a well-known antioxidant that has been used in trials individually or in combination with other drugs for the treatment of sexual dysfunction [18, 19]. Due to its antioxidant function, vitamin E plays a vital role in fighting various diseases, such as arteriosclerosis, oxidative stress, cancer, and cataracts [20]. Furthermore, topical Vitamin E used in menopausal women has been demonstrated to ease genitourinary syndrome and improve their sexual function. In this study, women used vaginal vitamin E suppositories (100 units) [19]. In another trial, vitamin E and ginseng supplements were used to enhance sexual performance in women aged 18 to 45. Women took a combination of 100 IU of vitamin E, 67 mg of Korean ginseng, and 40 mg of Siberian ginseng for six weeks. This supplement was better than the placebo only in increasing desire and satisfaction [21]. Nevertheless, a recent systematic review has yielded inconclusive evidence regarding the effect of vitamin E intake on the sexual function of women. The dose of vitamin E used for sexual dysfunction in the included trials ranged from 100 to 600 IU per day [22]. In the present trial, the dose of vitamin E was 50 mg.

Considering that there is no study on the simultaneous effect of saffron with vitamin E on sexual performance and considering that vitamin E may increase the effect of saffron, this study aimed to determine the effect of adding saffron to vitamin E on sexual function (primary outcome) and stress, anxiety, and depression (secondary outcomes) for eight weeks after starting the intervention and four weeks after the end of the intervention. Depression is one of the most important factors affecting sexual disorders [23–25]. Also, it has been shown that shorter sleep duration and higher insomnia scores were associated with decreased sexual function [26]. Therefore, the present study was conducted on women of reproductive age with sexual dysfunction and without comorbid sleep disorders or severe depression.

### Study hypotheses

1. Adding saffron to vitamin E improves overall sexual function and its subdomains in women of reproductive age.
2. Adding saffron to vitamin E improves the symptoms of depression, stress, and anxiety in women of reproductive age with sexual dysfunction.

## Methods

### Study design and participants

Our triple-blind randomized clinical trial received approval from the Ethics Committee of Tabriz University of Medical Sciences, with the assigned Ethics Code IR.TBZMED.REC.1399.120. Additionally, the trial was registered with the Iranian Registry of Clinical Trials under the code IRCT20100414003706N36. The study was conducted on a sample of women of reproductive age residing in Shiraz, Iran.

Inclusion criteria included married women aged 15 to 49 with at least a secondary school education, sexual dysfunction (FSFI score < 28), the ability to have regular sex with one’s partner based on the woman’s statement, the absence of severe or very severe depression (DASS-21 < 21), the absence of other known mental problems, the absence of sleep disorders (PSQI ≤ 5), the use of reliable contraception methods, and a lack of desire to become pregnant soon. The exclusion criteria included drug and alcohol addiction, sensitivity to saffron, pregnancy, the first six months after giving birth if the person was breastfeeding, receiving drug treatment for sexual dysfunction, temporary separation from one’s partner, the occurrence of significant stressors in the last three months, the presence of background diseases, the use of drugs that affect a person’s sexual response, premature menopause, daily consumption of saffron, daily consumption of vitamin E supplements, and participation in another trial.

### Sample size and sampling

The required number of samples was calculated to be 21 individuals per group using G*Power software, taking into account m1 = 23.4 (mean sexual function score) and SD_1_ = 3.5 based on the findings of Bahrami et al.‘s study [27], SD_1_ = SD_2_ = 3.5, and an increase of at least 15% in the mean sexual function score due to the intervention (m_2_ = 27.0), a 0.05 two-tailed type I error, and a test power of 90%. There were 25 people considered for each group, with an attrition rate of 15% being taken into account.

The SIB software program (Integrated Health System), employed in Iranian health centers, has registered the profiles of nearly 100% of the population residing in the city. Initially, the principal investigator used a random selection process to choose seven centers from a total of 25 comprehensive health service centers in Shiraz, Iran. The participant selection procedure involved extracting a list of women of reproductive age who were eligible for the study from the SIB computer program. Subsequently, the quota for each center was determined proportionally, and the initial samples were selected randomly from each center. Next, the study’s objectives were elucidated, and the individuals were invited to participate in the research via telephone. Prospective research participants underwent an initial screening process over the telephone to assess their eligibility based on predetermined inclusion and exclusion criteria. Those who met the initial eligibility criteria were subsequently invited to attend a briefing session at the health center.

Through face-to-face meetings, the study objectives and procedures were elucidated to the participants, and they signed written informed consent forms. Following this, participants completed a socio-demographic characteristics form, the Female Sexual Function Index (FSFI), the Depression, Anxiety, and Stress Scale (DASS-21), and the Pittsburgh Sleep Quality Index (PSQI). The study included individuals who displayed sexual dysfunction (FSFI score < 28), did not have severe or very severe depression (score < 21 on the DASS-21), and did not have sleep issues (PSQI score ≤ 5).

### Randomization and interventions

The allocation sequence was determined using stratified block randomization (stratified based on moderate or mild/normal depression severity) with a randomly varied block size of four and six and a 1:1 allocation ratio using a computer program. The experimental group received a 15 mg saffron capsule (safrotin) in the morning and a capsule containing 15 mg saffron and 50 mg vitamin E (safradide) in the evening. The control group received a placebo saffron capsule (apparently identical to safrotin) in the morning and a capsule containing 50 mg of vitamin E and placebo saffron (which was visually indistinguishable from safradide) in the evening. The pharmaceutical preparations, including the active drugs and the placebo, were formulated and provided by Green Plants of Life Pharmaceutical Company, Iran.

For each participant, a package containing four small envelopes containing either medication or placebo was prepared and numbered by the sequence of allocation. Each small envelope contained 30 capsules to be taken in the morning or evening for four weeks (two extra capsules in case of loss). The researcher sequentially opened the packages per the participants’ enrollment order. Subsequently, two small envelopes were dispensed to each participant for utilization in the morning and evening during the initial four-week period. Upon the conclusion of the fourth week and the required assessments, the second round of small parcels was dispatched. An individual who was not involved in the sampling and data collection process determined the allocation sequence and prepared drug packages. Blinding procedures were implemented to ensure that the participants, recruiters, allocators, data collectors, and analysts were unaware of the type of intervention administered to the participants.

Subsequent assessments were conducted at three time points: 4 and 8 weeks after administration of the pharmaceutical agents in a face-to-face setting and 4 weeks following cessation of drug use via remote communication channels such as phone or WhatsApp. During the in-person follow-up, the checklists of drug usage and associated side effects were gathered, along with the remaining medications. Additionally, the FSFI and DASS-21 questionnaires were re-administered. During the second monthly meeting, participants were given the questionnaires to be completed four weeks after they ceased consumption. They were instructed to complete and submit these questionnaires to the researcher via WhatsApp four weeks after the meeting. The study participants were subjected to two follow-up sessions within the initial month. Subsequently, monthly reminders were given via SMS or phone calls to ensure compliance with medication intake, completion of checklists and questionnaires, and meeting attendance.

### Measures

The present investigation employed several instruments, including socio-demographic and obstetric characteristics, FSFI, DASS-21, PSQI, and a checklist of medications and their associated side effects.

#### Socio-demographic and obstetric characteristics

It included personal and demographic characteristics of individuals (such as age, education level of wife and husband, occupation of wife and husband, length of the marriage, number of living children, method of contraception, smoking, hookah use, level of life satisfaction, exercise, and vitamin D consumption).

#### The female sexual function index (FSFI)

It is a standard questionnaire used to assess women’s sexual function over the previous four weeks. The questionnaire comprises 19 items and encompasses six distinct domains of sexual function, namely desire (two questions), arousal (four questions), lubrication (four questions), orgasm (three questions), satisfaction (three questions), and pain (three questions). Each answer is evaluated on a scale of 0–5 or 1–5. The composite scores for each domain are calculated by summing the scores derived from the questions within each respective domain and multiplying them by the corresponding coefficient (sexual desire = 0.6, sexual arousal and lubrication = 0.3, orgasm, satisfaction, and pain = 0.4). The sexual desire score ranges from 1.2 to 6, while satisfaction scores range from 0.8 to 6. Other domains are evaluated on a scale of 0 to 6 points. The aggregate score, derived from the six domains, would range from 2 to 36. Higher scores are indicative of better sexual function. Sexual dysfunction is defined as a total sexual function score of 28 or lower [28]. Mohammadi et al. [29] have verified the reliability and validity of the questionnaire’s Persian version in Iran.

#### The depression, anxiety, and stress scale-21 items (DASS-21)

This scale is a shortened version of the DASS-42, comprising three distinct subscales that measure stress, depression, and anxiety. The questionnaire comprises 21 questions, with seven items allocated to each of the three subscales. The scoring system is based on a Likert scale ranging from 0 (none applicable) to 3 (highly applicable). The score for each scale is determined independently, while the total score is not calculated. This questionnaire has been validated in Iran, and its validity and reliability have been confirmed [30].

#### Pittsburgh sleep quality index (PSQI)

This self-report questionnaire is the gold standard for assessing sleep quality and diagnosing adult sleep disorders. The tool comprises seven distinct components, each assigned a numerical score ranging from 0 to 3. The cumulative scores of the seven components constitute the overall score of the questionnaire, which ranges from 0 to 21. A score above 5 is indicative of poor sleep quality [31]. This questionnaire has been approved for use in Iran, and its validity and reliability have been established [32].

### Data analysis

After the data were collected, they were analyzed with SPSS 21 software. The Smirnov-Kolmogorov test was utilized to verify the normality of the distribution of quantitative data. The distribution of the variables of the age of the spouse, sleep quality score, sexual desire, sexual arousal, lubrication, satisfaction, pain, depression, and anxiety were found to be non-normal. Various statistical tests were employed to assess the socio-demographic characteristics of the experimental and control groups. The chi-square test was used for rank and nominal qualitative variables, the independent t-test for quantitative variables with a normal distribution, and the Mann-Whitney for quantitative variables without a normal distribution. Repeated measures (ANOVA and ANCOVA) were employed to compare the outcomes of the two groups in cases of normality of data distribution. When the data did not exhibit a normal distribution, the Mann-Whitney test was employed to compare the outcomes of the two groups, while the Wilcoxon test was utilized for within-group comparisons. The analyses were conducted on an intention-to-treat basis. A significance level of *p* > 0.05 was deemed significant.

## Results

**Fig. 1:**
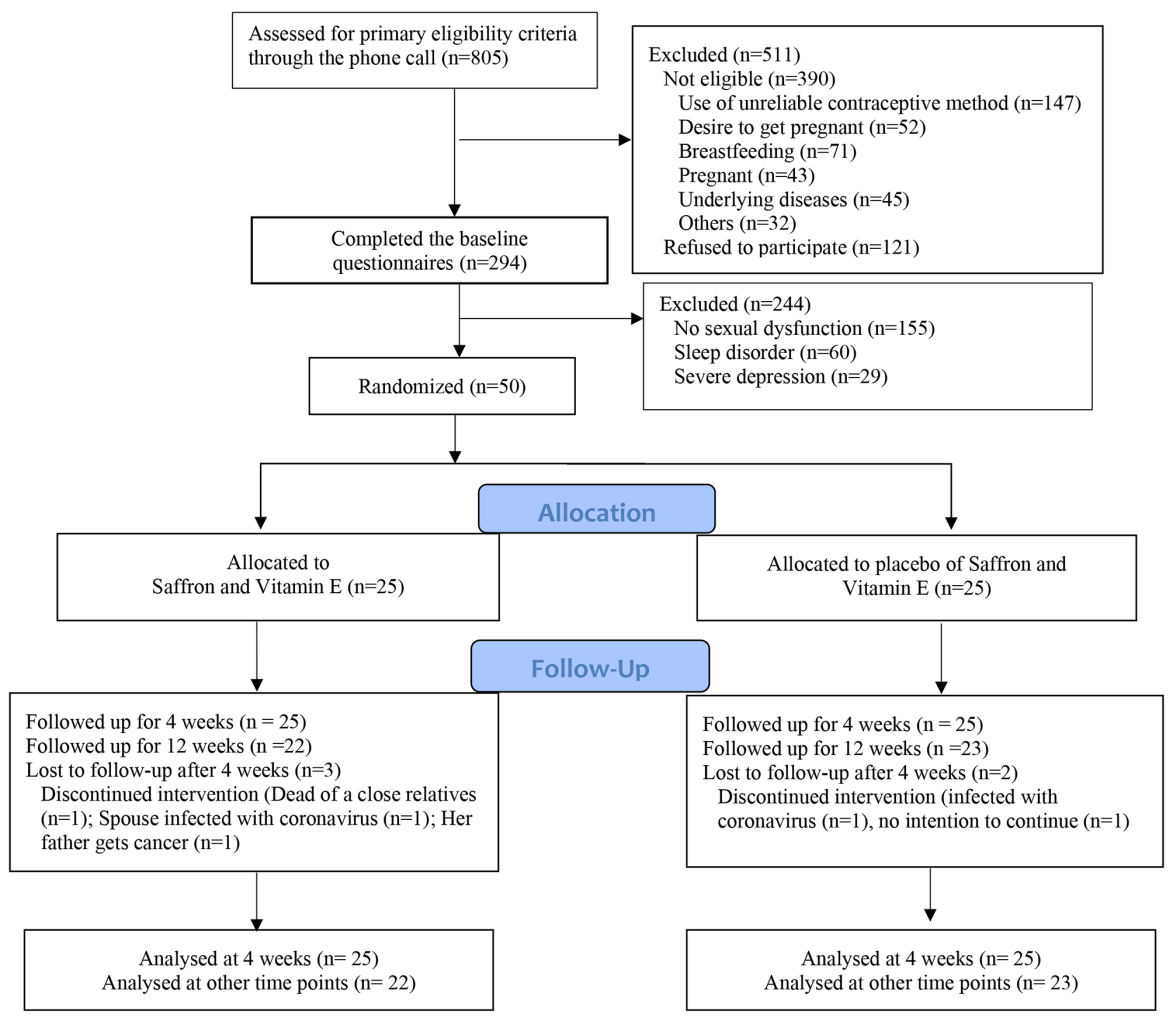
Flow diagram of the study

Enrollment of participants took place between August 2020 and February 2021, with follow-up ending in May 2021. Of the 813 individuals selected through the computer system, 121 declined to participate for various reasons, including fear of contracting coronavirus, employment obligations, and high workload. Additionally, 390 individuals did not meet the basic eligibility criteria. A total of 294 individuals completed the baseline questionnaires, namely, the FSFI, DASS, and PSQI measures. Of the total number of participants, 244 did not participate in the study for various reasons. Among these, 155 had no sexual dysfunction, 60 had sleep disorders, and 29 had both sleep disorders and depression. Of the 50 women included in the study, 25 were assigned to the saffron and vitamin E group (experimental group) and 25 to the vitamin E and saffron placebo group (control group). Only five individuals—three from the experimental group and two from the control group—were followed up until the end of the fourth week of the intervention. The remaining participants were followed up until the 12th week, which is four weeks after the intervention ended. Five women (three in the saffron and vitamin E group and two in the vitamin E and placebo group) were lost to follow-up after the first follow-up visit; the rest were followed up until the end of the trial (Fig. 1).

Of the five women lost, one in the intervention group had taken the prescribed capsules for only 14 days, and one in the control group for five days. Out of all the participants, just four individuals (two in each group) failed to adhere fully to the specified drug regimen. In the intervention group, one participant adhered to the drug regimen for 15 days, while another participant adhered for 12 days. In the control group, one participant adhered for 24 days, while another adhered for 13 days. One person reported headache in the intervention group, and one person reported dry mouth in the control group. No other side effects were reported by the participants.

Table 1 indicates that there were no significant differences between the two groups in terms of socio-demographic characteristics. These characteristics include the woman’s age (*p* = 0.860), husband’s age (*p* = 0.823), length of marriage (*p* = 0.462), body mass index (*p* = 0.085), sleep score (*p* = 0.497), woman’s education level (*p* = 0.188), husband’s education level (*p* = 1.00), marital satisfaction (*p* = 0.138), number of children (*p* = 0.088), contraceptive methods (*p* = 0.780), employment status (*p* = 0.529), non-smoking (*p* = 1.00), and not exercising (*p* = 0.157).

**Table 1:** Baseline characteristics of participants by the study groups

Variable	Saffron + Vitamin E (*n* = 25)	Placebo of saffron + Vitamin E (*n* = 25)	*P*
	Mean (SD^a^)	Mean (SD^a^)	
Age (years)	35.2 (6.1)	35.6 (6.6)	0.860 ^b^
Duration of marriage (years)	14.3 (7.5)	12.7 (7.7)	0.461 ^b^
Body Mass Index (kg/m2)	25.2 (2.5)	27.5 (4.0)	0.085 ^b^
(Percent)	Number (Percent)	Number	
Education			0.439^c^
Secondary school	3 (12%)	1 (4%)	
High school	11 (44%)	9 (36%)	
University	11 (44%)	15 (60%)	
Employed	6 (43%)	8 (57%)	0.529^d^
Spouse education			1.000^c^
Guidance	2 (8%)	1 (4%)	
High school	10 (40%)	10 (40%)	
University	13 (52%)	14 (56%)	
Number of children ≤ 2	16 (64%)	21 (84%)	0.226^d^
No Smoking	25 (100%)	25 (100%)	1.000 ^d^
Satisfaction with marriage			0.138 ^c^
Completely satisfied	2 (8%)	6 (24%)	
Satisfied	18 (72%)	16 (64%)	
Neither satisfied nor dissatisfied	5 (20%)	3 (12%)	
No regular exercise	22 (88%)	18 (72%)	0.247^d^

^a^ Standard Deviation; ^b^ Independent t test; ^c^ Chi-square for trend test; ^d^ Chi – square test

**Fig. 2:**
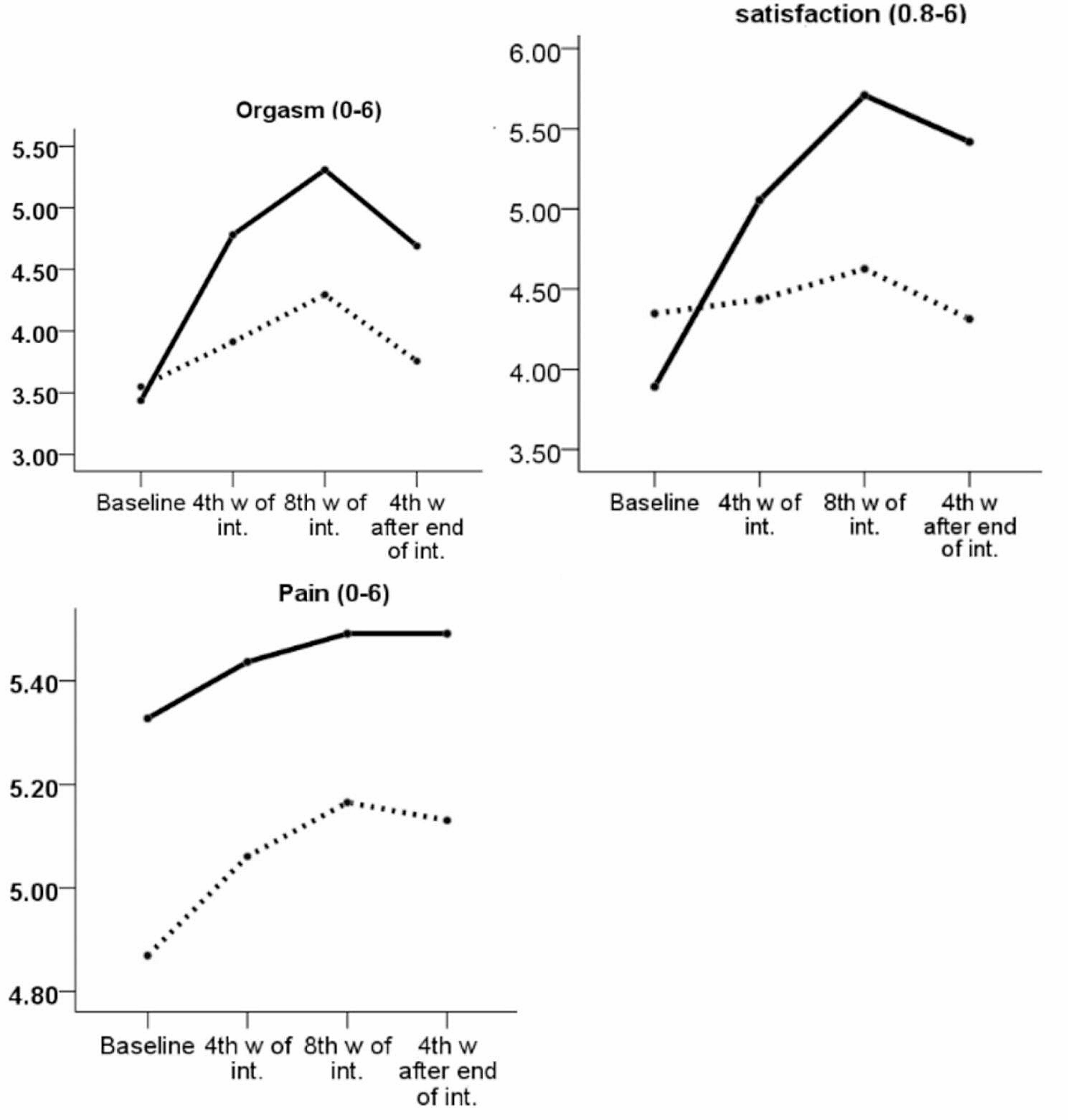
Sexual function scores at different time-points by study groups; Higher scores indicate the better sexual function

After adjusting the stratified factor, the ANCOVA test revealed no statistically significant difference between groups in terms of the overall score of sexual function at baseline (AMD: -1.3; 95% CI: -3.2 to 0.6; *p* = 0.177). The study employed a repeated measures ANOVA test to analyze the effect of the intervention on sexual function, controlling for the pre-intervention score and stratified factor. Additionally, the Sidak test was used to control for multiple comparisons. The results indicated that, overall, the experimental group had a significantly higher mean score for sexual function compared to the control group (AMD: 4.6; 95% CI: 3.1 to 6.1; *p* < 0.001) across all time points after the intervention (Table 2; Fig. 2).

**Table 2:** Comparison of total FSFI and stress among study groups

Variable	Saffron + Vitamin E (*n* = 23) Mean (SD^c^)	Vitamin E + Placebo of saffron (*n* = 22) Mean (SD^c^)	Mean difference (95% CI^d^); *P*
**Total FSFI (Score range: 2 to 36)**			4.6 (3.1 to 6.1); *p* < 0.001^b^
Baseline	22.6 (3.7)	24.0 (2.9)	-1.3 (3.2- to 0.6); *p* = 0.177^a^
4 weeks after intervention	28.3 (2.7)	25.1 (3.9)	3.9 (2.3 to 5.6); *p* < 0.001^a^
8 weeks after intervention	30.9 (2.1)	26.6 (3.6)	5.1 (3.7 to 6.4); *p* < 0.001^a^
4 weeks after the end of intervention	28.6 (3.2)	24.8 (4.0)	4.8 (3.2 to 6.5); *p* < 0.001^a^
**Stress (Score range: 0 to 21)**			-2.3 (-3.1 to -1.5); *p* < 0.001^b^
Baseline	7.8 (3.6)	8.3 (3.8)	-0.6 (-2.5 to 1.3); *p* = 0.544^a^
4 weeks after intervention	5.4 (2.3)	7.5 (3.0)	-1.7 (-2.7 to -0.7); *p* < 0.001^a^
8 weeks after intervention	3.8 (1.8)	6.8 (2.9)	-2.8 (-4.0 to -1.7); *p* < 0.001^a^
4 weeks after the end of intervention	4.8 (2.1)	7.4 (2.8)	-2.4 (-3.3 to -1.5); *p* < 0.001^a^

In the sexual function, higher scores indicate the better sexual function and in the stress, lower scores indicate the better situation

^a^ ANCOVA; ^b^ Repeated measures ANOVA; ^c^ Standard Deviation; ^d^ 95% Confidence Interval

The Mann-Whitney-U test was employed to compare the mean scores of sexual desire, arousal, orgasm, and satisfaction between the two groups. The findings revealed that the experimental group had significantly higher mean scores than the control group at all three time points (*p* < 0.001). In addition, only eight weeks after the intervention did the experimental group have a statistically significant higher mean lubrication score than the control group (*p* = 0.004). Nonetheless, no statistically significant difference was observed at the fourth week of the intervention (*p* = 0.053) and four weeks after the intervention ceased (*p* = 0.053). The statistical analysis revealed that there was no significant difference in the mean pain score between the experimental group and the control group at the time points of four (*p* = 0.556) and eight (*p* = 0.827) weeks after the commencement of the intervention and four weeks after the cessation of the intervention (*p* = 0.704) (Table 3; Fig. 2).

**Table 3:** Comparison of sexual function subscales, depression and anxiety among study groups

Variable	Saffron + Vitamin E (*n* = 23)		Vitamin E + Placebo of saffron (*n* = 22)		*P*-value^a^
	Mean (SD^b^)	Median [Per 25 to 75]	Mean (SD^b^)	Median [Per 25 to 75]	
**Desire** (Score range: 1.2 to 6)					
Baseline	2.7 (0.8)	2.4 [2.4 to 3.6]	3.3 (0.5)	3.0 [2.7 to 3.6]	0.043
4 weeks after intervention	3.9 (0.5)	3.6 [3.6 to 4.2]	3.4 (0.7)	3.6 [2.4 to 3.9]	< 0.001
8 weeks after intervention	4.5 (0.4)	4.8 [4.2 to 4.8]	3.8 (0.6)	3.6 [3.6 to 4.2]	< 0.001
4 weeks after the end of intervention	3.9 (0.6)	3.6 [3.6 to 4.3]	3.2 (0.7)	3.6 [2.4 to 3.6]	< 0.001
**Arousal** (Score range: 0 to 6)					
Baseline	3.1 (0.8)	3.3 [2.5 to 3.6]	3.4 (0.6)	3.3 [3.0 to 3.6]	0.385
4 weeks after intervention	4.4 (0.5)	4.5 [3.9 to 4.8]	3.6 (0.7)	3.6 [3.1 to 4.0]	< 0.001
8 weeks after intervention	4.9 (0.6)	5.1 [4.8 to 5.4]	3.9 (0.6)	3.9 [3.6 to 4.5]	< 0.001
4 weeks after the end of intervention	4.4 (0.6)	4.6 [3.9 to 4.8]	3.5 (0.6)	3.6 [3.3 to 3.9]	< 0.001
**Lubrication** (Score range: 0 to 6)					
Baseline	4.1 (0.8)	4.2 [3.6 to 4.6]	4.5 (1.3)	4.5 [3.1 to 5.5]	0.424
4 weeks after intervention	4.7 (0.8)	4.5 [3.9 to 5.4]	4.7 (1.3)	4.8 [3.4 to 5.4]	0.053
8 weeks after intervention	5.0 (0.7)	4.9 [4.5 to 5.5]	4.8 (1.1)	5.4 [4.2 to 5.7]	0.004
4 weeks after the end of intervention	4.8 (0.8)	4.8 [3.9 to 5.4]	4.8 (1.2)	5.4 [3.9 to 6.0]	0.053
**Orgasm** (Score range: 0 to 6)					
Baseline	3.4 (1.0)	3.6 [2.8 to 4.2]	3.5 (0.8)	3.6 [2.8 to 4.0]	0.937
4 weeks after intervention	4.8 (0.6)	4.8 [4.4 to 5.2]	3.9 (1.0)	4.0 [3.0 to 4.6]	< 0.001
8 weeks after intervention	5.3 (4.0)	5.4 [5.2 to 5.6]	4.3 (0.9)	4.4 [4.0 to 4.8]	< 0.001
4 weeks after the end of intervention	4.7 (0.7)	5.0 [4.0 to 5.2]	3.7 (0.9)	3.6 [3.2 to 4.8]	< 0.001
**Satisfaction** (Score range: 0 to 6)					
Baseline	3.9 (1.0)	4.0 [3.2 to 4.8]	4.3 (0.8)	4.4 [3.6 to 4.8]	0.197
4 weeks after intervention	5.0 (0.7)	4.8 [4.8 to 5.4]	4.4 (0.8)	4.8 [3.6 to 4.8]	< 0.001
8 weeks after intervention	5.7 (0.5)	6.0 [5.2 to 6.0]	4.6 (0.8)	4.8 [4.4 to 4.8]	< 0.001
4 weeks after the end of intervention	5.4 (0.8)	5.8 [4.8 to 6.0]	4.3 (0.9)	4.4 [3.6 to 4.8]	< 0.001
**Pain** (Score range: 0 to 6)					
Baseline	5.3 (0.9)	6.0 [4.4 to 6.0]	4.9 (1.1)	5.2 [4.2 to 6.0]	0.281
4 weeks after intervention	5.4 (0.7)	6.0 [4.8 to 6.0]	5.1 (0.9)	4.8 [4.8 to 6.0]	0.556
8 weeks after intervention	5.5 (0.7)	6.0 [4.8 to 6.0]	5.2 (0.9)	5.6 [4.4 to 6.0]	0.827
4 weeks after the end of intervention	5.5 (0.8)	6.0 [5.1 to 6.0]	5.1 (1.0)	5.2 [4.8 to 6.0]	0.704
**Depression** (Score range: 0 to 21)					
Baseline	4.6 (2.8)	5.0 [3.0 to 6.5]	3.7 (2.4)	3.0 [2.5 to 6.0]	0.142
4 weeks after intervention	2.5 (1.7)	3.0 [2.0 to 4.0]	3.1 (2.0)	3.0 [2.0 to 4.5]	0.011
8 weeks after intervention	1.6 (1.5)	2.0 [0.0 to 3.0]	3.0 (2.3)	3.0 [1.0 to 4.0]	0.005
4 weeks after the end of intervention	2.4 (1.8)	2.0 [1.0 to 4.0]	3.5 (2.4)	3.0 [2.0 to 5.0]	0.007
**Anxiety** (Score range: 0 to 20)					
Baseline	4.1 (2.6)	4.0 [2.0 to 6.0]	4.7 (3.3)	4.0 [2.0 to 6.0]	0.799
4 weeks after intervention	2.0 (1.5)	1.0 [1.0 to 3.0]	3.9 (3.0)	3.0 [2.0 to 6.0]	0.016
8 weeks after intervention	1.5 (1.3)	1.0 [0.7 to 2.2]	3.3 (2.8)	3.0 [1.0 to 4.0]	0.177
4 weeks after the end of intervention	1.5 (1.6)	1.0 [0.0 to 2.0]	4.1 (3.4)	3.0 [2.0 to 5.0]	0.002

For the sexual function subscales, higher scores indicate the better sexual function and for the anxiety and depression, lower scores indicate the better situation

^a^ Mann Whitney U; ^b^ Standard Deviation

**Fig. 3:**
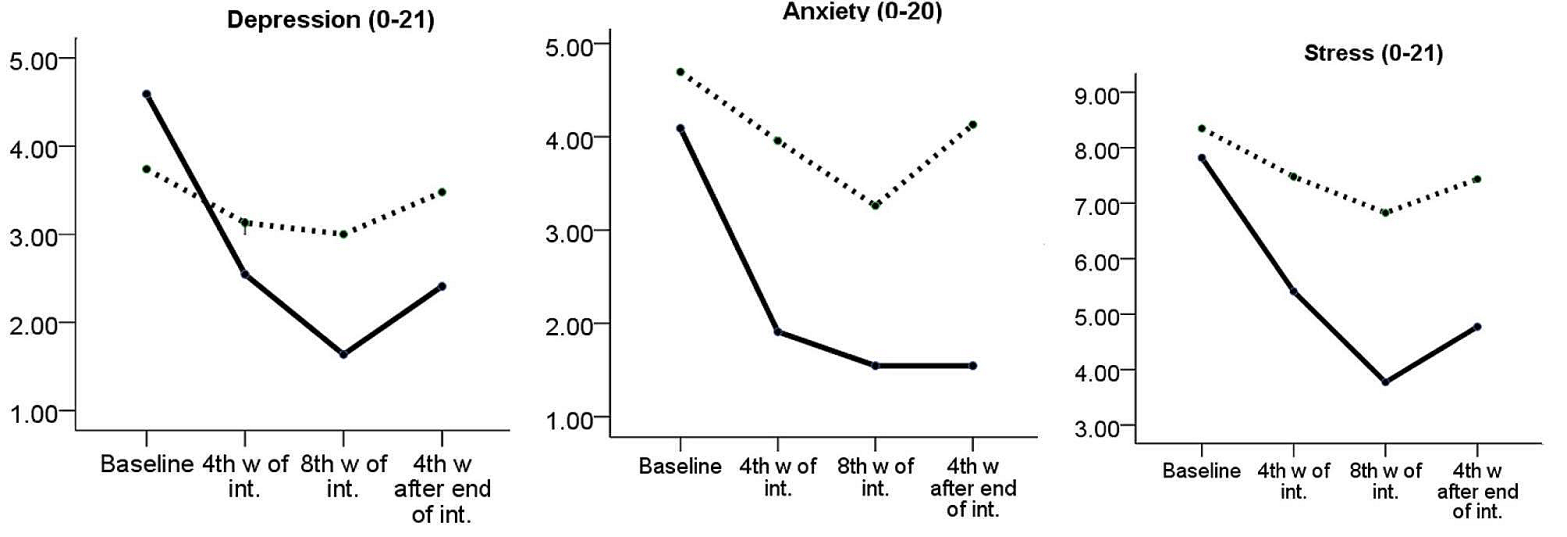
Depression, anxiety and stress scores at different time-points by study groups; Lower scores indicate the better situation

The Mann-Whitney U test was utilized to compare the two groups based on their average depression and anxiety scores. The findings indicated that the experimental group had a significantly lower mean depression score than the control group at all three time points, namely, four (*p* = 0.011) and eight (*p* = 0.005) weeks after the start of the intervention and four weeks after the end of the intervention (*p* = 0.007). The findings indicated that the mean anxiety score of the experimental group was significantly lower than that of the control group at four weeks after the intervention (*p* = 0.016) and four weeks after the intervention was completed (*p* = 0.002). No statistically significant difference was observed between the two groups in terms of the average anxiety score eight weeks following the initiation of the intervention (*p* = 0.177) (Table 3; Fig. 3).

A repeated measures ANOVA was used to analyze the effect of the intervention on stress levels while controlling for the pre-intervention score and quota factor. It was found that the experimental group’s mean stress score after the intervention was significantly lower than the control group (AMD: -2.3; 95% CI: -3.1 to -1.5; *p* < 0.001) (Table 2; Fig. 3).

## Discussion

The current study found a significant difference between vitamin E plus saffron and vitamin E plus placebo in terms of the primary outcome (total score of sexual function and its subscales, excluding pain) and the secondary outcomes (depression, anxiety, and stress).

The present study showed the positive effect of saffron plus vitamin E on the total score of sexual function, which is consistent with the results of previous studies on women aged 18 to 39 without considering the state of depression [11], postmenopausal women [12], depressed women taking fluoxetine [13], and a systematic review and meta-analysis involving 173 male and female participants [10]. Women with severe and very severe depression were excluded from the study in the current investigation. However, the intervention significantly reduced the average scores for stress, anxiety, and depression. Therefore, it is possible that the effect of saffron on improving sexual function in this study was at least partially attributable to the enhancement of the mental state of the research participants. Systematic reviews also demonstrate that saffron has a beneficial effect on people’s mental health, including the reduction of anxiety and depression [9, 33]. Even its effectiveness in treating depression is thought to be comparable to that of fluoxetine [34]. Given that the saffron-consuming group had a significantly high score for sexual function four weeks after the intervention began and that the research tool (the FSFI) looks at sexual function over the previous month, it can probably be concluded that saffron has an immediate effect on sexual function. Prior studies have also demonstrated such an immediate effect [11, 12]. The fact that sexual function was reported to improve eight weeks after the intervention than it was four weeks later suggests that the effect likely grows stronger the longer it is used. These findings are in line with those of another study that looked at sexually dysfunctional women of reproductive age. In that study, the frequency of disorders was reported to be 91% and 66%, respectively, in the group receiving saffron 4 and 8 weeks after the start of the intervention and 100% in the group receiving placebo at both time points [11]. To our knowledge, no trials have been conducted to examine the effect of using this intervention for longer than eight weeks on sexual function.

The decline in sexual function that began four weeks after the intervention was stopped and reached its peak about four weeks after the intervention began most likely indicates a gradual loss of effect. Longer follow-ups are required in order to determine when the saffron effect is completely lost.

Consistent with the findings of one previous study on women of reproductive age [11], our findings show that saffron significantly improves desire, arousal, lubrication, orgasm, and satisfaction, but not pain. Saffron significantly improved all subscales, including pain, in a trial involving postmenopausal women [12]. The observed discrepancy may be attributed to the comparatively lower mean baseline pain score (indicating higher pain intensity) during sexual activity among postmenopausal women in contrast to their reproductive-age counterparts in this investigation.

The current study’s findings regarding saffron’s beneficial effect on depression, anxiety, and stress align with prior research [34]. However, our study did not include women with severe or very severe depression. However, the intervention significantly reduced the depression score. Several systematic reviews have demonstrated the favorable effect of saffron on enhancing individuals’ mental health, specifically depression and anxiety [9, 33].

In this study, we tried to minimize participant non-cooperation (due to fear of contracting the coronavirus) and to encourage them to answer sensitive and private questions accurately by establishing a good relationship with the participants, interviewing them in a private room by making appointments, and assuring them of the confidentiality of the information collected. We also tried to minimize selection, performance, and detection biases by considering all the principles of clinical trials, including randomization, allocation concealment, and blinding of participants and all individuals involved in participant recruitment and allocation, data collection, and analysis. We also believe that attrition bias in this study was low due to the absence of loss to follow-up during the first-month follow-up, the low number and similar reasons for loss to follow-up after the first month in the groups, as well as the similarity of the effect of intervention in the first month in two analyses with and without excluding people without full follow-up. Moreover, the follow-up period of this study was longer than in previous similar studies, as it included follow-ups four weeks after the discontinuation of the drug, which can be considered a strength of this study. However, longer follow-ups are recommended to determine the effects of prolonged use and the long-term effects after discontinuation. The random selection of samples from the whole city can be considered another strength of this study, as it increases the generalizability of the results. However, the relatively high frequency of individuals with no initial access or an unwillingness to participate in the study, as well as the relatively high eligibility criteria, may have negatively affected the generalizability of the results of this study to all women of childbearing age. In this study, we were not able to evaluate the interaction effect of saffron and vitamin E on the outcomes because we did not have a control group that included vitamin E placebo and saffron placebo. Such an effect could be assessed in future trials.

## Conclusions

Using the combination of saffron and vitamin E is more effective in improving sexual function and its domains compared to vitamin E alone in women of reproductive age with sexual dysfunction without severe depression. Also, it diminishes the degree of depression, anxiety, and stress more compared to vitamin E alone. Therefore, due to the high prevalence of sexual disorders in women and the safety of this combination, its use is recommended as a suitable option for the treatment of sexual disorders in this group of women.

## Data Availability

The datasets generated and/or analysed during the current study are not publicly available due to limitations of ethical approval involving the patient data and anonymity but are available from the corresponding author on reasonable request.

## Abbreviations

FSFI: Female Sexual Function Index
DASS-21: Depression, Anxiety, Stress Scale
PSQI: Pittsburgh Sleep Quality Index
ANOVA: Analysis of Variance
ANCOVA: Analysis of Covariance
AMD: Adjusted mean difference
SD: Standard Deviation
